# Contactless Camera-Based Sleep Staging: The HealthBed Study

**DOI:** 10.3390/bioengineering10010109

**Published:** 2023-01-12

**Authors:** Fokke B. van Meulen, Angela Grassi, Leonie van den Heuvel, Sebastiaan Overeem, Merel M. van Gilst, Johannes P. van Dijk, Henning Maass, Mark J. H. van Gastel, Pedro Fonseca

**Affiliations:** 1 Department of Electrical Engineering, Eindhoven University of Technology, 5612 AZ Eindhoven, The Netherlands; 2 Sleep Medicine Center Kempenhaeghe, 5591 VE Heeze, The Netherlands; 3 Philips Research, 5656 AE Eindhoven, The Netherlands

**Keywords:** remote photoplethysmography, heart rate variability, pulse rate variability, sleep stage classification, contactless monitoring

## Abstract

Polysomnography (PSG) remains the gold standard for sleep monitoring but is obtrusive in nature. Advances in camera sensor technology and data analysis techniques enable contactless monitoring of heart rate variability (HRV). In turn, this may allow remote assessment of sleep stages, as different HRV metrics indirectly reflect the expression of sleep stages. We evaluated a camera-based remote photoplethysmography (PPG) setup to perform automated classification of sleep stages in near darkness. Based on the contactless measurement of pulse rate variability, we use a previously developed HRV-based algorithm for 3 and 4-class sleep stage classification. Performance was evaluated on data of 46 healthy participants obtained from simultaneous overnight recording of PSG and camera-based remote PPG. To validate the results and for benchmarking purposes, the same algorithm was used to classify sleep stages based on the corresponding ECG data. Compared to manually scored PSG, the remote PPG-based algorithm achieved moderate agreement on both 3 class (Wake–N1/N2/N3–REM) and 4 class (Wake–N1/N2–N3–REM) classification, with average κ of 0.58 and 0.49 and accuracy of 81% and 68%, respectively. This is in range with other performance metrics reported on sensing technologies for wearable sleep staging, showing the potential of video-based non-contact sleep staging.

## 1. Introduction

While sleep is something natural for many of us, it is not the case for everyone. For some, it will be necessary to obtain more detailed and objective information on sleep structure, to assess any potential sleep disorder. The gold standard to measure sleep is polysomnography (PSG). Although PSG is widely used in clinical practice and in research, it has some drawbacks. PSGs are typically performed in (specialized) sleep centres using many wired sensors measuring multiple physiological parameters, such as cortical activity, cardiac activity and breathing. During these measurements patients may feel unfamiliar with the environment, and cables may feel obtrusive. Besides these shortcomings that influence actual sleep quality [1,2], in-lab (video) PSG is rather costly, and analysis is complex. Therefore, it is rarely used for sleep assessment over consecutive nights and may be insufficient to unearth infrequent events, or night-to-night variability, resulting in a less representative picture of sleep [2]. A less obtrusive way of measuring sleep could alleviate these issues, especially in populations who are less tolerant to PSG, such as children or patients with intellectual disabilities [3].

Sleep stages are traditionally scored by analysing the electroencephalogram, electrooculogram and electromyogram signals that are part of the PSG measurement. Extensive research has been conducted to obtain surrogate measures of sleep and sleep stages staging using other, less obtrusive, techniques. A frequently used and proven technique is by measuring heart rate variability (HRV). Different HRV metrics indirectly reflect autonomic nervous system activity, which in turn are known to express sleep stages; as such, it has been used to identify sleep stages with reasonable accuracy [4,5,6]. The gold standard sensor for measuring cardiac activity is the electrocardiogram (ECG), a wired setup which is obtrusive as well, especially during sleep. Recent advances in sensor technology and data analysis techniques facilitated research on alternative and less obtrusive sleep monitoring setups based on HRV [7]. Wrist-worn devices for measuring photoplethysmography (PPG) and actigraphy have shown promise in this area [8,9,10,11,12]. However, these devices still require contact with the body of the subject being measured and suffer, albeit to a lesser extent, of issues related to comfort and obtrusiveness. Camera-based monitoring is a promising example of contactless estimation of vital signs that are generally part of a sleep assessment [13]. Previous work showed the monitoring of vital signs during sleep, focusing on the detection and estimation of body movements and body posture [14,15], heart rate [16,17], respiration [14,16,18,19], or blood oxygen saturation [16], all without requiring any cables or sensors connected to the patient and in near darkness. Researchers have applied the monitoring of body movements directly towards the automated classification of sleep stages [20]. Others used estimated respiratory movements to detect characteristic features of different sleep stages [21] and respiratory events during sleep [18]. To the best of our knowledge, no previous work was carried out on camera HRV-based monitoring of sleep stages.

The purpose of this study is to evaluate, in near darkness, a camera-based, remote PPG setup for the contactless measurement of pulse rate variability and automated classification of sleep stages in healthy subjects. The system makes use of measured pulse-to-pulse HRV and a previously developed sleep staging algorithm based on HRV features. Results of the estimated sleep stages are compared with sleep stages manually scored from a simultaneously recorded PSG.

## 2. Methods

### 2.1. Experimental Protocol

The system was evaluated on the HealthBed-cohort of healthy adults (aged 18 or above). Exclusion criteria were: a presence of any previously diagnosed sleep disorders, a Pittsburgh Sleep Quality Index larger than 5, an Insomnia Severity Index larger than 7, a Hospital Anxiety and Depression Scale score larger than 8, shift-work, pregnancy, use of any medication, except for birth control medicine, or any clinically relevant neurologic or psychiatric disorders or other somatic disorders that could influence sleep or limit the ability to adhere to the study procedures. All participants were recruited via online advertisements. A total of 96 participants were enrolled into the HealthBed study and scheduled for an overnight sleep registration at the Sleep Medicine Centre Kempenhaeghe, Heeze, The Netherlands. Of these, 51 participants were recorded with the camera-based remote PPG setup. Due to technical problems in the measurement setup software during the recordings, the system failed to record the whole night of five participants, and the data of these participants are excluded from further analysis. The remaining participants (*n* = 46) had an average age of 35.3 years (±14.5 years), 34 females and 12 males.

In addition to the measurement setup, a reference PSG was acquired with a Grael 4K PSG amplifier (Compumedics Limited, Victoria, Australia). PSG sensors were set up according to the AASM guidelines and sleep stages were visually scored in 30-s epochs by an experienced sleep technician, all according to the AASM manual for scoring of sleep and associated events (Version 2.2, July 2015) [22]. The manually scored sleep stages were used as a reference to evaluate the performance of the sleep staging algorithm.

### 2.2. Measurement Setup

**Figure 1:**
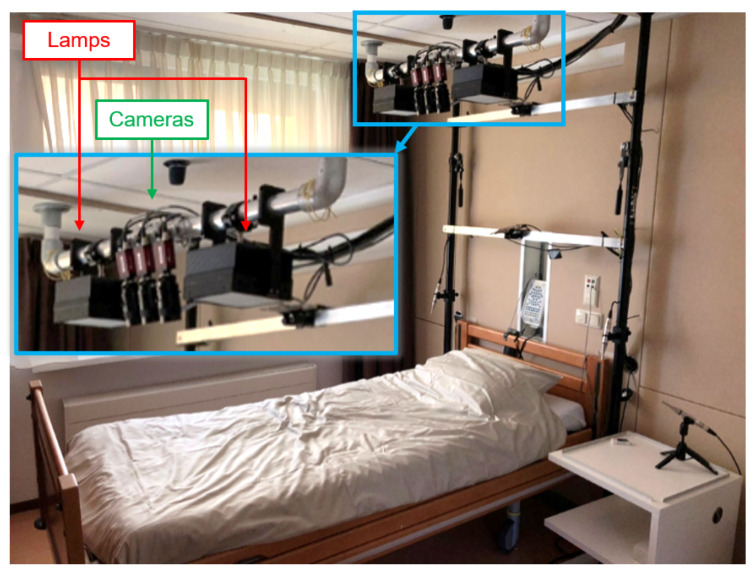
Measurement setup. Three cameras positioned between two illumination units were placed above the bed to continuously capture the patient’s face during the overnight recording.

The camera-based remote PPG setup comprised cameras and dedicated infrared illumination mounted on a bar located above the head of the participant (Figure 1). This viewpoint was selected because of skin visibility, from which the cardiac pulse signal can be extracted. To suppress influences of distortions (e.g., motion and noise) on the pulse detection, three horizontally spaced, identical monochrome CCD cameras (AVT Manta G-283B, Allied Vision GmbH, Stadtroda, Germany) with zoom lenses (M6Z1212-3S, Computar, Tokyo, Japan) were equipped with different optical band-pass filters to obtain spectral selectivity in near-infrared. The centre wavelengths of these filters were 760, 800 and 890 nm, wavelengths that are almost invisible to the human eye. The zoom factor of the lenses was selected to cover the full width of the bed, to alleviate limitations on sleeping position. Two identical custom-developed illumination units were placed on both sides of the cameras. These units consist of LEDs with three wavelengths matching the filters of the cameras. An optical diffuser was placed in front of the LEDs to improve the spectral homogeneity of the illumination area. The impact of lightning on each participant’s experience was evaluated after the nocturnal recording by means of a questionnaire (Appendix A).

The cameras were simultaneously triggered by the video acquisition system. Frames were stored uncompressed at a resolution of 968 × 728 pixels and transferred via ethernet. To limit needed data storage and ensure a stable frame rate, frame rate was set to 15 Hz. Synchronization of the video and PSG data was achieved by generating a synchronization signal by the video acquisition system and sampling this signal simultaneously with the PSG amplifier during PSG acquisition.

### 2.3. Camera-Based Remote PPG, Pulse Extraction and Inter-Pulse Interval Detection

The HRV-based sleep staging algorithm requires an accurate estimation of the time distance between consecutive heart beats (inter-pulse intervals, IPI, from the remote PPG setup). To enable accurate pulse-to-pulse duration estimations from the remote PPG setup, we used a previously developed processing framework to extract the pulse signal from the raw video data [23]. This framework considers different lying positions and possible occlusions and was validated in a laboratory setting on a limited dataset. In short, the framework started with pre-processing the raw video by dividing the frames into subregions using a box-shaped interpolation kernel. Traces were generated by temporal concatenation of the intensity values for each subregion and for all three wavelengths. Next, all traces were bandpass filtered (0.6–3 Hz, corresponding to a heart rate between 36 and 180 beats per minute), and pulse signals were extracted using a robust pulse extraction method [24]. By similarity mapping and using the pulse signal as a feature, a distinction was made between living and non-living pixels. All pulses from the detected living pixels were combined to reconstruct a single pulse signal. Next, the pulse signal (15 Hz) was resampled at 240 Hz using a cubic spline interpolation method and rescaled in amplitude between 0 and 1. Finally, systolic peaks were detected using an automatic multiscale-based peak detection method that does not need any additional user input prior to analysis [25]. No form of post-processing or pruning of the detected peaks was performed. The time between two consecutive peaks (i.e., IPI) was calculated and only inter-pulse intervals with a maximum of 1.5 s (corresponding to a heart rate of 40 beats/min) and a minimum of 0.3 s (200 beats/min) were assumed to be valid; all others were excluded.

### 2.4. Sleep Stage Classifier

A previously developed machine learning algorithm for automatic sleep staging was used to classify sleep stages [6,26]. The classifier, trained with 132 HRV features extracted from ECG interbeat-interval data, consists of 1 dense layer, 3 bidirectional LSTM layers, 2 dense layers and an output with softmax for four classes: wake, N1/N2, N3, and REM. As in previous work [6], the classifier was pre-trained with 584 recordings (of 195 healthy sleepers and 97 sleep disordered patients) of the SIESTA dataset [27], using as training ground-truth PSG consensus scorings of three experienced sleep technicians. None of the recordings acquired in the present study were used to tune or in any way optimize the sleep staging algorithm or the input HRV features. The sleep stage classifier was used, without modifications, to classify sleep stages based on the same input HRV features extracted from the IPI signal obtained with the remote PPG setup. For each participant, a simplified four-class hypnogram was obtained for the “lights off” period of the recording.

### 2.5. Sleep Staging Performance

To evaluate the sleep staging algorithm, the hypnogram obtained with the remote PPG setup was compared against the manually scored sleep stages based on PSG. Since the classifier outputs a simplified hypnogram, the labels of the manually scored sleep stages N1 and N2 were combined in a single “N1/N2” class, while the remaining classes (wake, N3, and REM) were used without changes. Cohen’s kappa coefficient of agreement (or κ) and accuracy were used to describe epoch-per-epoch agreement between the predictions obtained with each setup, and the manually scored hypnogram from the gold standard PSG. The classifier was further evaluated in a three-class classification task, by merging the N1/N2 and N3 labels of the prediction and ground-truth reference in a single non-REM (NREM) class. In addition, the classification performance for each sleep stage was determined, by considering each class separately against the merged remaining classes; in this case, in addition to κ and accuracy, also sensitivity, specificity, and PPV were calculated.

### 2.6. ECG Benchmark-Sleep Staging and Pulse Detection Performance

For benchmarking purposes, the same classifier was also used to classify sleep stages based on the inter beat intervals (IBIs) derived from the ECG Lead II channel included in the PSG recording of each participant. The ECG signal, recorded at 512 Hz, was first high-pass filtered to remove baseline wander using a linear phase filter using a Kaiser window of 1.016 s, a cut-off frequency of 0.8 Hz and a side-lobe attenuation of 30 dB [28]. QRS complexes were detected using a Hamilton–Tompkins QRS detector and further localized with a post-processing algorithm [29]. The time distance between consecutive R-peaks was used to calculate the resulting IBI time series.

The performance indicators of the sleep staging classifier based on ECG with respect to manually scored sleep stages were compared to the performance indicators using remote PPG. A Wilcoxon signed rank test was used to detect significant differences (at a *p*-value of 0.05) between both.

The detected heart beats from the ECG signal, and derived IBI time series were used as a ground truth to evaluate the pulse detection performance of the remote PPG setup. Each detected pulse was matched with beats detected in the ECG signal in the corresponding PSG recording. A match was defined when a pulse was found within a range of 250 ms of a single QRS complex, in which case the pulse was marked as true positive (TP). If a pulse could not be matched to any QRS complex, it was marked as false positive (FP). If multiple pulses were matched to one QRS complex, one pulse was considered as TP and the remaining as FP. All QRS complexes that were not matched with any pulses are considered false negatives (FN). The coverage of the inter-pulse time series of the remote PPG setup was calculated as the sum of all IPIs related to duration of the PSG measurement. The pulse detection performance was evaluated by calculating the sensitivity and positive predictive value (PPV). The bias (i.e., mean time difference between all matched pulses and their corresponding ECG beats) and trigger jitter (i.e., the standard deviation of these differences) were calculated as well as the percentage of pulses within 50 ms and 100 ms of the closest QRS complex, as in [30,31], for the correctly detected pulses according to the criteria used to calculate coverage and sensitivity, and after correcting for the average localization error (bias) of each participant. Finally, parameters describing the resemblance between the IPI and IBI time series, such as root mean square error (RMSE) and Pearson’s correlation coefficient (ρ), were calculated.

Furthermore, we evaluated whether pulse detection performance explains the differences in sleep staging performance of both setups (i.e., remote PPG vs. ECG). The difference in performance between both setups was obtained, per participant, by calculating the difference in κ obtained with the remote PPG setup ($\kappa_{remotePPG}$) and with ECG ($\kappa_{ECG}$) i.e., $\kappa_{ECG}$ – $\kappa_{remotePPG}$. Spearman’s correlation coefficients were used to evaluate possible relations between the difference in sleep stage classification performance and pulse detection performance metrics. Since the test was repeated 9 times (once per metric), a Bonferroni correction for repeated tests was used. For that reason, we used as a threshold for significance a *p*-value of 0.006 or below.

## 3. Results

### 3.1. Questionnaire Results on Experienced Obtrusiveness

None of the participants reported any substantial influence of the camera-based remote PPG measurement setup (i.e., light source and/or camera’s) on their sleep beyond the effect of the conventional PSG setup (see Table A1 in Appendix A), for more detailed results on the questionnaire).

### 3.2. Sleep Stage Classifier and Performance

**Figure 2:**
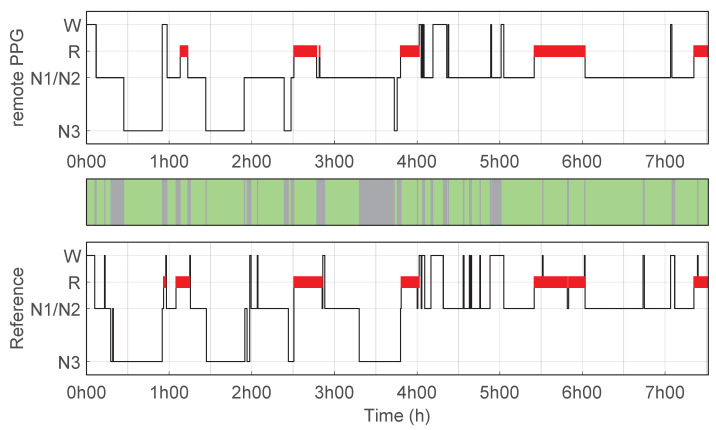
Hypnogram for the highest performance of remote PPG setup. Hypnograms for one example recording of a participant for which the predictions of the remote PPG setup compared to PSG manual scoring has the highest $\kappa_{remotePPG}$ value (accuracy 82%, κ = 0.71); (**top**) hypnogram predictions using remote PPG; (**bottom**) reference hypnogram based on PSG manual scoring. The coloured bar in between illustrate the correspondence with the reference hypnogram, with green indicating that the prediction of each setup matches the reference, and grey indicating a mismatch for that epoch.

**Figure 3:**
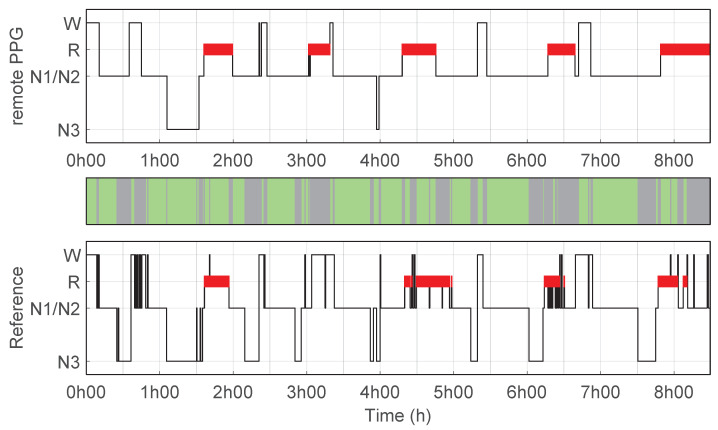
Hypnogram for an average performance of remote PPG setup. Hypnograms for one example recording of a participant for which the predictions of the remote PPG setup compared to PSG manual scoring has $\kappa_{remotePPG}$ closest to the average performance (accuracy 67%, κ = 0.46); (**top**) Hypnogram predictions using remote PPG; (**bottom**) reference hypnogram based on PSG manual scoring.

Figure 2 illustrates an example of a hypnogram predicted using the remote PPG setup as well as the reference hypnogram based on PSG manual scoring. This example corresponds to the hypnogram with the highest $\kappa_{remotePPG}$ for the predicted sleep stages (4 classes) using the remote PPG setup compared to manual scoring. Figure 3 illustrates another example, this one for the hypnogram for which the remote PPG setup had the $\kappa_{remotePPG}$ closest to the average performance.

Table 1 shows the sleep staging performance of the classifier, the agreement between the manual scored PSG stages (i.e., reference) and the predicted sleep stages based on the remote PPG setup. The highest performance is obtained for REM detection, with an average $\kappa_{remotePPG}$ of 0.61. Confusion matrices for all predictions of the remote PPG setup against the reference are shown in Appendix B.

### 3.3. ECG Benchmark-Sleep Staging and Pulse Detection Performance

**Figure 4:**
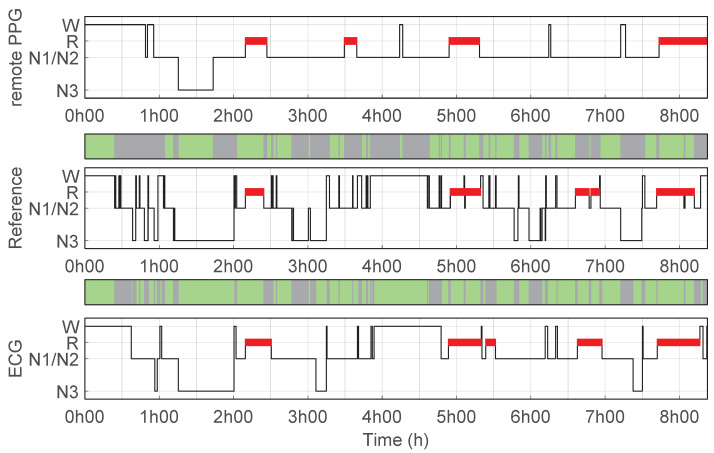
Hypnogram with a higher wake classification sensitivity with ECG than with Remote PPG. Hypnograms for one example recording of a participant where ECG achieved a higher wake classification sensitivity than with remote PPG; (**top**) Hypnogram predictions using remote PPG, accuracy 55%, $\kappa_{remotePPG}$ = 0.34 vs. reference PSG; (**middle**) reference hypnogram based on PSG manual scoring; (**bottom**) hypnogram predictions using ECG, accuracy 75% $\kappa_{ECG}$ = 0.65 vs. reference PSG.

**Figure 5:**
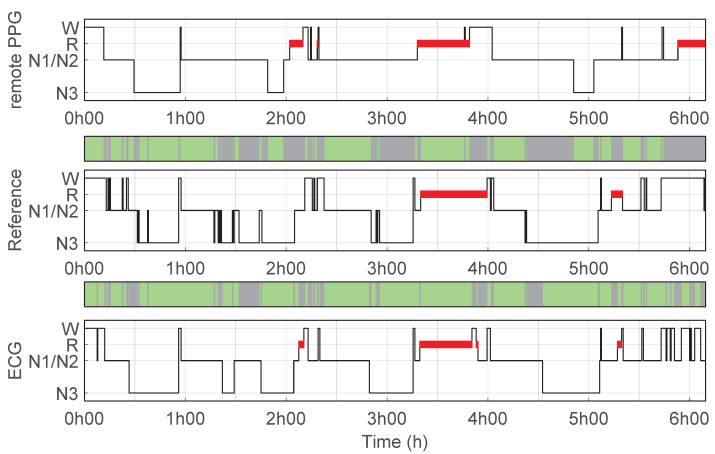
Hypnogram with a higher N3 classification sensitivity with ECG than with Remote PPG. Hypnograms for one example recording of a participant where ECG achieved a higher N3 classification sensitivity than with remote PPG; (**top**) Hypnogram predictions using remote PPG, accuracy 58%, $\kappa_{remotePPG}$ = 0.39 vs. reference PSG; (**middle**) reference hypnogram based on PSG manual scoring; (**bottom**) hypnogram predictions using ECG, accuracy 80% $\kappa_{ECG}$ = 0.71 vs. reference PSG.

The originally ECG trained classification algorithm showed overall higher performance compared to the performance of the remote PPG setup. Only the specificity of classifying Wake and N3 (two-class binary tasks) were not significantly different compared to the performance of the remote PPG setup. However, for these classes, the sensitivity using ECG was substantially and significantly higher than with the remote PPG setup. Figure 4 and Figure 5 illustrate examples of two recordings where these sensitivity differences are visible. Respectively, the figures frequently show misclassification by the remote PPG setup of sleep stages that were manually scored as Wake and N3, and which were generally classified correctly using ECG. A full overview of the performance of classifier based using ECG data are presented in Appendix C.

Table 2 indicates the pulse detection performance of the remote PPG setup in comparison with ECG. The coverage of IPI time series of the remote PPG setup was on average 83.1%, a minimum of 63.3% and a maximum of 91.5%. The pulse detection algorithm achieved a relatively high mean sensitivity of 83.1% ± 6.9, and PPV of 96.6% ± 3.9 compared to ECG. Even in participants where the coverage and sensitivity is low, the localization is very accurate, with a minimum and average of 86.2% and 94%, respectively, of the correctly detected pulses found within 100 ms of the corresponding QRS complex.

The difference in sleep stage classification performance ($\kappa_{ECG}$ – $\kappa_{remotePPG}$) per participant was significantly correlated with the sensitivity of pulse detection within the same participant (ρ = −0.45, *p* = 0.002), and with the correlation between IPIs (with remote PPG) and IBIs (with ECG) (ρ = −0.52, *p* < 0.001). In both cases, a lower pulse detection performance was related to a larger difference between the classification performance of both setups on a per participant level, with the classification using ECG outperforming the remote PPG setup.

## 4. Discussion

We evaluated a contactless system for the automated classification of sleep stages in a group of 46 healthy participants, based on the measurement of instantaneous pulse rate using a camera-based remote PPG setup. HRV based sleep stages were compared with manual scored sleep stages based on the gold-standard video PSG and showed a good performance on automated sleep stages classification with an average κ = 0.58 and an accuracy of 81% on 3-class sleep staging and an average κ = 0.49 and an accuracy of 68% on 4-class sleep staging. This unique system enables, under near darkness, a contactless assessment of sleep stages, offering a major advantage over current practice (i.e., polysomnography) as well as wearable solutions, which are frequently experienced as obtrusive and influencing normal sleeping behaviour. None of the participants reported any substantial influence of the camera-based remote PPG setup on their sleep.

To put the performance of our method in some context, we compared our results with previously published work on wearable and nearable devices for sleep staging as summarized by Imtiaz et al. [7]. It should be noted that performance estimates in this overview are not directly comparable, as studies applied a variety of other sensors and validated in different cohorts. It does show, however, that both accuracy and κ of our remote PPG setup are in range with performance metrics reported in other studies [7]. For 3-class sleep staging with multiple sensing modalities (including ECG, EEG, PPG, actigraphy), Imtiaz et al. [7] reported an average κ = 0.50 (range: 0.27–0.74) and an average accuracy of 76% (range: 70–85%), which is a lower performance compared to our results. For similar 4-class classification strategies (Wake-N1/2-N3-REM), Imtiaz et al. reported an average κ = 0.54 (range: 0.36–0.72) and an average accuracy of 72% (range: 57–90%) which is slightly better compared to our results.

The proposed camera-based remote PPG setup can measure pulse rate variability in a reliable, contactless, and unobtrusive manner, which in turn can be used to obtain a relatively accurate representation of sleep and sleep stages. This may be particularly important for patient populations who do not tolerate the traditional wired systems or even wearable solutions, such as sleep disordered patients, children, or patients with intellectual disabilities. Furthermore, this setup could also be used for patient monitoring in settings with a high patient throughput, such as the general ward of a hospital, in which physical contact between the monitoring technology and the patients should be limited to reduce the chances of cross-contamination and spreading of diseases. Future studies should validate the camera-based remote PPG setup in these different populations and settings.

The sleep staging algorithm used to classify sleep stages from the remote PPG setup was previously trained on ECG recordings. To validate the performance and for benchmarking purposes, the algorithm was used to classify sleep stages using corresponding ECG data as well. The performance of the classifier based on the ECG input (κ = 0.65, accuracy: 77.4%, see Appendix C) is at the expected level for healthy participants. The same classification algorithm was tested before on HRV and body movement data of sleep disordered patients where it showed a comparable performance (κ = 0.60, accuracy: 75.9%) [6]. As might be expected, using a different cardiac sensing modality (remote PPG) than the one used to train the sleep staging algorithm (ECG) led to a decrease in performance. Previous work showed a similar phenomenon, in that case using an ECG trained algorithm on PPG data [32]. However, when the algorithm was adapted to the characteristics of a different sensor [33] or even completely retrained on device specific data (e.g., PPG data), the classification performance of the algorithm increased to levels similar to the ECG baseline [9]. It can be expected that using similar strategies on larger datasets of camera-based remote PPG measurements would lead to a similar increase in the performance of the algorithm.

As a rule of thumb, supported by the results of Imtiaz et al. [7] and Selvaraju et al. [13], the further away from the body sensing is performed, the more difficult it seems to correctly detect the precise temporal location of heart beats and consequently to correctly classify sleep stages based on heart rate variability. Comparing both setups, the largest performance differences were found while classifying Wake and N3. Although both setups exhibit a lower N3 sensitivity when compared with other sleep stages, this seems worse when using the remote PPG setup. A possible explanation for this might be the lower initial sample frequency of the remote PPG setup (i.e., 15 Hz). This may introduce errors when estimating HRV metrics that are known to require higher temporal resolution needed, for example, to express the higher frequency characteristics of parasympathetic nervous system tone in IBI series, in turn needed to correctly distinguish between light (N1/N2) and deep sleep (N3). In future work, a higher temporal resolution should be preferred for a more accurate estimation of the inter-pulse times. On the other hand, the misclassification of Wake might be related to the fact that the remote PPG setup always generates a plausible pulse signal, even in cases when the signal-to-noise ratio is very low because of the participant moving during wakefulness periods, or when an insufficient amount of skin is visible. In these cases, this may result in the attempt to classify unreliable inter-pulse variations, resulting in incorrect sleep stage predictions. A possible, but not yet implemented solution, might be to use a signal quality indicator. Using the camera input, this detector should be able to detect the presence of enough skin area, followed by the detection of large body movements which can serve as a surrogate measure of actigraphy and facilitate the detection of Wake [23].

A limitation of remote PPG monitoring pertains to a fundamental aspect of the monitoring principle itself. The setup needs enough visible skin (high enough signal-to-noise ratio) to be able to estimate a pulse signal. A possible solution would be to use optical zooming, to increase the number of skin pixels and therefore the signal-to-noise ratio. However, while sleeping, people unintentionally roll over or pull the blanket over their body, thus covering part of their skin. A multi camera setup or an automated camera aiming setup might be necessary to keep enough visible skin pixels after zooming.

## 5. Conclusions

HRV-based sleep staging by a non-contact remote PPG setup holds potential and deserves further research. In future studies, the representation of sleep and sleep stages assessed using remote PPG setup could be combined with other parameters that can be obtained with camera-based techniques, such as limb movements, or body movements caused by respiration. In addition, the remote PPG setup could be combined with other contactless monitoring techniques, for example other camera- or radar-based techniques to assess and estimate heart rate, respiration, body movements, and even blood oxygen saturation levels (i.e., SpO2) [7,13,16,22,34] during sleep. This would not only allow the direct comparison of different techniques, but likely would increase sleep staging performance of sleep staging and add resilience by redundance. Eventually, combining such techniques may allow a fully contactless and unobtrusive assessment and classification of sleep, as well as sleep-related disorders such as sleep apnea.

## Figures and Tables

**Table 1:** Classification performance using remote PPG compared with the reference, i.e., manual scoring. Mean (SD) per class.

	$\kappa_{\mathrm{remotePPG}}$ (−)	Accuracy (%)	Sensitivity (%)	Specificity (%)	PPV (%)
2 classes ${}^{a}$					
Wake	0.42 (0.21)	89.7 (7.6)	44.7 (21.7)	95.4 (7.2)	59.2 (23.1)
N1/N2	0.43 (0.15)	71.5 (7.4)	80.8 (9.3)	62.3 (10.6)	68.1 (10.1)
N3	0.48 (0.15)	86.4 (4.6)	43.7 (18.1)	98.1 (2.2)	85.5 (17.4)
REM	0.61 (0.16)	88.2 (5.1)	76.8 (16.0)	90.8 (5.7)	64.0 (17.6)
3 classes ${}^{b}$	0.58 (0.14)	81.0 (7.5)			
4 classes ${}^{c}$	0.49 (0.13)	67.9 (8.7)			

^*a*^ 2 classes, one versus the rest, where the single class was considered as the ‘positive’ class and the others are together the negative class. ^*b*^ 3 classes: Wake, N1/N2/N3 (Non-REM), and REM. ^*c*^ 4 classes Wake, N1/N2, N3, and REM.

**Table 2:** Pulse detection performance of the remote PPG setup, in comparison with ECG.

Parameter (Unit)	Mean	SD	Min.	Max.	Correlation with $\kappa_{\mathrm{ECG}}-\kappa_{\mathrm{remotePPG}}$
Coverage (%)	83.1	6.9	63.3	92.4	ρ = −0.32, *p* = 0.028
*Pulse detection*					
Sensitivity (%)	84.3	7.3	61.9	94.2	ρ = −0.45, *p* = 0.002
PPV (%)	96.6	3.9	82.5	99.6	ρ = −0.21, *p* = 0.156
*Pulse timing*					
Bias (ms)	35.4	26.7	−20.0	108.7	ρ = 0.24, *p* = 0.113
Trigger jitter (ms)	54.9	8.8	37.7	75.0	ρ = 0.33, *p* = 0.025
Pulses within 50 ms of QRS (%)	68.3	7.4	49.3	84.2	ρ = −0.25, *p* = 0.092
Pulses within 100 ms of QRS (%)	94.0	3.6	86.2	99.0	ρ = −0.32, *p* = 0.029
*Pulse interval*					
RMSE (ms)	70.6	23.6	36.2	156.4	ρ = 0.30, *p* = 0.040
ρ (−)	0.75	0.13	0.40	0.94	ρ = −0.52, *p* < 0.001

**Figure A1:**
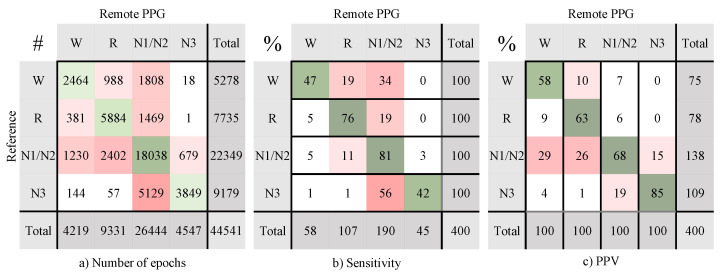
Confusion matrices for remote PPG versus reference PSG. (**a**) number of epochs, with a total of 30,235 (68%) correctly classified (diagonal, in green); (**b**) sensitivity, relative to ground truth class; (**c**) PPV, Positive predictive value, relative to predicted class.

**Figure A2:**
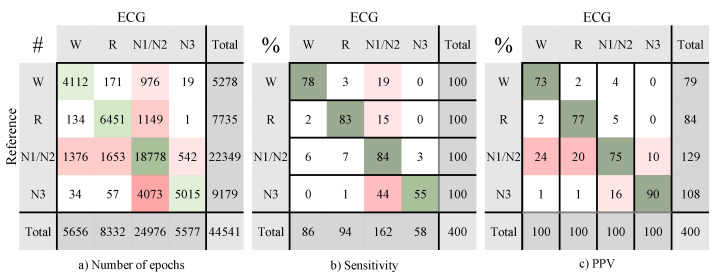
Confusion matrices for ECG versus reference PSG. (**a**) number of epochs, with a total of 34,356 (77%) correctly classified (diagonal, in green); (**b**) sensitivity, relative to ground truth class; (**c**) PPV, positive predictive value, relative to predicted class.

## Data Availability

The data used in this study are available from the Sleep Medicine Centre Kempenhaeghe upon reasonable request. The data can be requested by presenting a scientific research question and by fulfilling all the regulations concerning the sharing of the human data (e.g., privacy regulations). The details of the agreement will depend on the purpose of the data request and the entity that is requesting the data (e.g., research institute or corporate). Each request will be evaluated by the Kempenhaeghe Research Board and, depending on the request, approval from independent medical ethical committee might be required. Specific restrictions apply to the availability of the data collected with cameras not comprised in the standard PSG set-up, and unprocessed data are not publicly available because of data privacy protection of participants. As the cameras are used under license, processed data are not publicly available. These data are, however, available from the authors upon reasonable request and with permission of the licensors.

## Abbreviations

The following abbreviations are used in this manuscript:

AASM	American Academy of Sleep Medicine
ECG	Electrocardiogram / Electrocardiography
FN	False Negative
FP	False Positive
HRV	Heart Rate Variability
IBI	Inter Beat Interval
IPI	Inter Pulse Interval
N1/2/3	Sleep stages 1, 2 and 3 with No Rapid Eye Movements
PPG	Photoplethysmography
PPV	Positive Predictive Value
PSG	Polysomnography
QRS	QRS-complex, the combination of three of the graphical deflections seen on a typical ECG
REM	Sleep stage with Rapid Eye Movement
RMSE	Root Mean Square Error
TP	True Positive
κ	Cohen’s kappa coefficient of agreement
$\kappa_{ECG}$	Agreement between the predicted sleep stages using the ECG setup and the reference
$\kappa_{remotePPG}$	Agreement between the predicted sleep stages using the remote PPG setup and the reference

## Appendix A. Questionnaire Results

The morning after each polysomnography recording, participants were asked to answer the following three questions, on a five-point Likert scale (1—Very much; 2—A lot; 3—A little bit; 4—Hardly; 5—Not at all):

1. Did the presence of the remote PPG measurement setup influence your consideration to participate in this study?
2. Did the presence of the camera influence your sleep?
3. Did the presence of the additional light-source, of the remote PPG setup, influence your sleep?

As a result, a total of 43 out of 46 participants completed the questionnaire. Table A1 shows the answers, summarized per question.

**Table A1:** Results of the questionnaire on the perceived obtrusiveness of the remote PPG setup, totals per answer option.

	1: Very Much	2: A Lot	3: A Little Bit	4: Hardly	5: Not at All
Question 1	-	-	3	7	33
Question 2	-	-	6	10	27
Question 3	-	1	1	8	33

## Appendix B. Confusion Matrices Remote PPG Setup

Figure A1 illustrates the confusion matrices with aggregated performance results for the remote PPG setup compared to reference PSG manual scoring for a grand total of 44,541 epochs, from 46 participants.

## Appendix C. ECG Performance

Table A2 shows the sleep staging performance of the classifier, the agreement between the manual scored PSG stages (i.e., reference) and the predicted sleep stages based on ECG analysis. Furthermore, Figure A2 illustrates the confusion matrices with aggregated performance results for the remote PPG setup compared to reference PSG manual scoring for a grand total of 44,541 epochs, from 46 participants.

**Table A2:** Classification performance using ECG compared with the reference, i.e., manual scoring. Mean (SD) per class.

	$\kappa_{\mathrm{ECG}}\left(-\right)$	Accuracy (%)	Sensitivity (%)	Specificity (%)	PPV (%)
2 classes ${}^{a}$					
Wake	0.70 (0.13)	93.9 (2.7)	79.3 (16.2)	95.9 (3.4)	72.9 (17.1)
N1/N2	0.56 (0.12)	78.4 (6.1)	83.7 (7.0)	72.8 (9.3)	74.8 (8.8)
N3	0.61 (0.15)	89.6 (4.2)	56.0 (18.1)	98.5 (1.9)	89.7 (14.9)
REM	0.73 (0.10)	93.1 (4.0)	84.0 (12.8)	95.1 (3.1)	77.4 (12.0)
3 classes ${}^{b}$	0.74 (0.10)	87.6 (4.3)			
4 classes ${}^{c}$	0.65 (0.10)	77.5 (6.5)			

${}^{a}$ 2 classes, one versus the rest, where the single class was considered as the ‘positive’ class and the others are together the negative class. ${}^{b}$ 3 classes: Wake, N1/N2/N3 (Non-REM), and REM. ${}^{c}$ 4 classes Wake, N1/N2, N3, and REM.
